# In vitro permissiveness of bovine neutrophils and monocyte derived macrophages to *Leishmania donovani* of Ethiopian isolate

**DOI:** 10.1186/s13071-016-1441-5

**Published:** 2016-04-18

**Authors:** Geremew Tasew, Endalamaw Gadisa, Adugna Abera, Aboma Zewude, Menberework Chanyalew, Abraham Aseffa, Markos Abebe, Uwe Ritter, Ger van Zandbergen, Tamás Laskay, Ketema Tafess

**Affiliations:** Ethiopia Public Health Institute, Leishmaniasis Research Laboratory, P.O. Box 1242, Addis Ababa, Ethiopia; Armauer Hansen Research Institute, Leishmaniasis Research Laboratory, P.O. Box 1005, Addis Ababa, Ethiopia; Department of Immunology, University of Regensburg, Franz-Josef-Strauß-Allee 11, D-93042 Regensburg, Germany; Paul-Ehrlich-Institute, Federal Institute for Vaccines and Biomedicines, Langen, Germany; Institute for Medical Microbiology and Hygiene, University of Lübeck, Ratzeburger Allee 160, D-23560 Lübeck, Germany; Department of Medical Laboratory Science, College of Health Sciences, Arsi University, P.O. Box 193, Asella, Ethiopia

**Keywords:** Bovine PMN, MDM, Zebu, Holstein Friesian, Reservoir host

## Abstract

**Background:**

Epidemiological studies in Ethiopia have documented that the risk of visceral leishmaniasis (VL, Kala-azar) is higher among people living with domestic animals. The recent report on isolation of *Leishmania donovani* complex DNA and the detected high prevalence of anti-leishmanial antibodies in the blood of domestic animals further strengthen the potential role of domestic animals in the epidemiology of VL in Ethiopia. In mammalian hosts polymorphonuclear cells (PMN) and macrophages are the key immune cells influencing susceptibility or control of *Leishmania* infection. Thus to substantiate the possible role of cattle in VL transmission we investigate the permissiveness of bovine PMN and monocyte derived macrophages (MDM) for *Leishmania* (*L.*) *donovani* infection.

**Methods:**

Whole blood was collected from pure Zebu (*Boss indicus*) and their cross with Holstein Friesian cattle. *L. donovani *(MHOM/ET/67/HU3) wild and episomal green fluorescent protein (eGFP) labelled stationary stage promastigotes were co-incubated with whole blood and MDM to determine infection of these cells. Engulfment of promastigotes by the cells and their transformation to amastigote forms in MDM was studied with direct microscopy. Microscopy and flow cytometry were used to measure the infection rate while PCR-RLFP was used to confirm the infecting parasite.

**Results:**

*L. donovani* infected bovine whole blood PMN in the presence of plasma factors and all cellular elements. Morphological examinations of stained cytospin smears revealed that PMN engulfed promastigotes. Similarly, we were able to show that bovine MDM can be infected by *L. donovani*, which transformed to amastigote forms in the cells.

**Conclusions:**

The in vitro infection of bovine PMN and MDM by *L. donovani* further strengthens the possibility that cattle might serve as source of *L. donovani* infection for humans.

## Background

Leishmaniases are neglected tropical diseases caused by obligate intracellular protozoan parasites and are endemic in 98 countries, the majority of which are developing countries, with more than 350 million people at risk globally [1, 2]. The three main clinical forms of leishmaniases are: cutaneous leishmaniasis (CL), mucocutaneous leishmaniasis (MCL) and visceral leishmaniasis (VL or Kala-azar). Among these forms, VL is a systemic disease and fatal if left untreated. It is caused by the *Leishmania* (*L.*) *donovani* complex in East Africa and the Indian subcontinent and by *Leishmania infantum* in Europe, North Africa and Latin America [3, 4]. More than 90 % of global VL cases occur in six countries: India, Bangladesh, Sudan, South Sudan, Ethiopia and Brazil [1, 5]. Up to approximately 0.4 million new cases of VL have been estimated to occur per year [1]. In addition, apart from malaria, it is the most common parasitic disease and accounts for more than 50,000 deaths each year [6, 7].

Based on the source of infection, VL transmission is basically grouped as zoonotic and anthroponotic where transmission of *Leishmania* (*L.*) *donovani* infection has been considered as anthroponotic in endemic countries such as East Africa [2]. However, *L. donovani* infected wild and domestic animals were reported in several foci [2, 8, 9]. Epidemiological reports also indicated the increased risk of VL in humans living in close proximity to domestic animals [2, 8]. Additional studies have emerged which strengthen the notion that domestic animals may serve as parasite hosts or, possibly, as reservoirs for human *L. donovani* VL. A study from Nepal [8] detected VL parasite DNA in domestic animals like cows, buffaloes, and goats. They also showed that proximity of humans to goats in particular constituted the highest risk factor for human infection. Furthermore, a recent study in Northwest Ethiopia detected *L. donovani* DNA from cattle and reported a positive correlation between anti-*Phlebotomus orientalis (P. orientalis)* saliva, and anti-*L. donovani* IgGs in cows, goats, and sheep [9]. In East Africa, *P. orientalis* is considered as the major vector for VL transmission [2].

Though parasitic DNA and antibodies against *L. donovani* were detected in domestic animals such as cattle, no study has yet demonstrated the presence of intact *L. donovani* parasites in vivo or in vitro in bovine cells in which these obligate intracellular parasites would be predicted to reside [10]. Therefore, we examined whether neutrophils and macrophages in bovine whole blood could be infected when exposed to live *L. donovani* promastigotes; such an observation would provide additional evidence supporting the hypothesis that domestic animals may serve as reservoir host for *L. donovani*. The infection rates of  *L. donovani* in PMN and MDM were compared among whole blood of indigenous Zebu and exotic Holstein-Zebu cross-breeds.

## Methods

### *Leishmania* parasite and culture

*L. donovani* (MHOM/ET/67/HU3) [9, 11] wild and episomal green fluorescent protein (eGFP) labelled, were donated by Prof. Dr. Ger van Zandbergen, Paul-Ehrlich-Institute, Federal Institute for Vaccines and Biomedicines, Langen, Germany. The wild type promastigotes were cultured in RPMI 1640 supplemented with 10 % FCS, 100U/100ug/ml penicillin streptomycin and 2 mM L-glutamine. Hygromycin-B (sigma), 30 μg/ml, was supplemented for cultivation of eGFP labelled parasites [12].

### Whole blood collection

Whole blood was collected in EDTA tubes from pure Zebu/*Boss indicus* (*n* = 6) and their cross with Holstein Friesian (*n* = 6). All animals were females, age of 6–8 years, and all tested negative for *Leishmania* exposure using immunochromatographic rapid diagnostic test (IT Leish, Bio-Rad). Whole blood cell count (WBC) and differential count for neutrophils and monocytes were performed using an automated hematology analyzer (Sysmex XT-1800i, Kobe, Japan).

### Whole blood and *L. donovani* stationary promastigote co-incubation

Whole blood (100 μl) was distributed in 1.5 ml eppendorf tubes and stationary phase promastigotes of *L. donovani* were added at 1x10^6^/100 μl. The tubes were then incubated in water bath at 37 °C for 22 h. Red blood cells were lysed by adding 500 μl 1x BD FACS lysing solution (BD Bioscience, USA) for 15 min at room temperature. The tubes were then centrifuged at 1000 xg for 8 min at room temperature, and then washed with 500 μl PBS (pH 7.2) with repeat centrifugation. After the supernatant was discarded, the cells were re-suspended in 400 μl PBS (pH 7.2). Cytospin smears from the cell suspension were stained with 10 % Giemsa staining solution and examined for PMN infection. Infection rate was calculated from the number of infected PMN per 200 PMN cells.

### Bovine peripheral blood mononuclear cells (PBMC) isolation

PBMC were isolated as previously described [13]. Isolated PBMCs were re-suspended in RPMI1640 medium supplemented with 100U/100ug/ml of penicillin/streptomycin, 10 % fetal bovine serum (FBS) and 2 mM L-glutamine (complete RPMI1640 medium).

### Bovine monocyte differentiation to macrophage

Isolated PBMCs were washed and re-suspended in polystyrene culture flasks containing complete RPMI 1640 medium with 1 % autologous plasma, and incubated at 37 °C, 5 % CO_2_ for 1.5–2 h. The non-adherent cells were discarded and tubes were washed twice with pre-warmed sterile PBS (pH 7.2) containing 5 % complete medium (wash media). Cells were then suspended in 5 ml of complete RPMI 1640 medium and incubated at 37^o^C, 5 % CO_2_. Cell washing and complete RPMI 1640 medium replacement was repeated every two days up to 6–8 days. After 6–8 days of culture, cell were washed twice with pre-warmed sterile PBS and kept on ice for 30 min with gentle agitation to detach the majority of adherent cells. Additional adherent cells were removed by a cell scraper and 91–97 % of cells were viable when assessed by trypan blue exclusion.

The detached cells were washed twice with wash media and centrifugation at 200 × g for 10 min. Finally monocytes derived macrophages (MDM) were re-suspended in 5 ml of complete RPMI 1640 medium on ice.

### MDM infection with *L. donovani* stationary stage promastigotes

Viable MDMs were seeded in flat bottomed 24-well culture plates (Nalge nunc international, Denmark), 16 well chamber slides (Thermo Scientific™ Nunc™ Lab-Tek™, USA) and 1.5 ml eppendorf tubes in complete RPMI 1640 culture medium. The cultures were then incubated for 30 min at 37 °C, 5 % CO_2_ to allow cell adherence. *Leishmania donovani* stationary promastigotes (wild type and eGFP labelled) were added to the cultures at a multiplicity of infection of 10 promastigotes to 1 MDM, and centrifuged at 200 xg for 5 min to promote contact. The plates were incubated for an additional 90 min at 37 °C, 5 % CO_2_ and then washed carefully with warm sterile wash medium until extracellular promastigotes were removed, and then further washed with pre-warmed PBS for 2–3 times and air dried. The plates were fixed with absolute methanol for 5 min and stained with 10 % Giemsa staining solution for 10–15 min and examined under a binocular microscope. Tubes containing MDM infected with eGFP labelled *L. donovani* was analyzed using a BD FACS Calibur flow cyotometry to measure the infection rate. At each infection time points, tubes were washed carefully with warm sterile wash medium until extracellular promastigotes were removed and a drop of the suspension was visualized under the microscope to confirm that extracellular parasites were removed from each tube.

### Co-incubation of infected whole blood cells with MDM

Bovine whole blood (100 μl) was distributed in 1.5 ml eppendorf tubes and 1x10^6^/100 μl stationary phase promastigotes of *L. donovani* were added and incubated for 22 h in a 37 °C water bath. Red blood cells were then lysed using a sterile 155 mM NH_4_Cl (ammonium chloride) solution for 15 min at room temperature, centrifuged at 1000 xg for 8 min and washed twice. Finally, pellets were re-suspended in 400 μl of RPMI complete medium. One hundred micro liters of the suspension was added to 24 well micro-plates (Nalge nunc international, Denmark) and chamber slide wells containing MDM. These were kept in a humidified incubator at 37 °C, 5 % CO_2_ for up to 16 days. Half of the plates were examined microscopically after staining with a 10 % Giemsa staining solution, while the other half were kept in culture at 37 °C, 5 % CO_2._

### Culturing *L. donovani* parasites from infected MDM

At the 16^th^ day of culture, *L. donovani* infected MDM (as described above) were washed with warm wash medium and examined under an inverted microscope for any motile extracellular parasites. Then supernatants were replaced by 300 μl of liquefied Novy-MacNeal-Nicolle (NNN) medium or 300 μl complete RPMI 1640 medium and cultures were incubated at 26 °C. The cultures were then examined under an inverted microscope every day for possible appearance of motile promastigotes.

### Polymerase chain reaction (PCR)-restriction fragment length polymorphism (RFLP) for species determination

Promastigotes cultured from in vitro infected bovine MDM were harvested by centrifugation at 1000 × g for 10 min at 4 °C. The genomic DNA was extracted by using Qiagen DNeasy for blood and tissue kits (QIAGEN GmbH, Germany) according to manufacturer’s protocol. The extracted DNA was quantified using a spectrophotometer. Cultured stationary phase promastigotes of reference strains: *L. aethiopica* (MHOM/ET/72/L100), *L. donovani* (MHOM/IN80/DD8), *L. infantum* (MHOM/FR/LEM-75), *L. tropica* (MHOM/SU/74/K27) and *L. major* (MHOM/SU/73/5-A SKH) were extracted using the same kit. Confirmation of the *Leishmania* species was achieved by PCR amplification of the ribosomal internal transcribed spacer-1 (ITS-1) sequences and RFLP analysis as described before [14]. In brief, LISTR/L5.8S primer pairs were used at 1 μl of each primer (15 pmol) with 12.5 μl HotStar Taq Master Mix and 2 μl (20 ng/μl of DNA) in a total volume of 25 μl. The amplification conditions were 30 cycles at 94 °C for 30 s, at 50 °C for 30 s, and at 72 °C for 30 s. The ITS-1-amplicon was then digested with the restriction enzyme *Hha*I. The reaction mix contained 17 μl PCR product, 2 μl of 10× enzyme buffer and 1 μl of restriction enzyme, and after vortexing was incubated for 4 h at 37 °C [15]. The PCR products and RFLP fragments were visualized by a trans-illuminator.

### Ethical issues

Ethical approval was obtained from the institutional review board of the Armauer Hansen Research Institute, and consent was obtained from the owners of the domestic animals (Sebeta Agro Industry Farm) for the collection of blood samples by a veterinarian. International animal experimentation guidelines were followed.

## Results

### Complete blood cell and differential count

Prior to the infection experiments, white blood cell count (WBC) and differential cell counts for neutrophils and monocytes were analyzed from whole blood of the animals. The mean total WBC count (± standard error of the mean, SEM) per μl of blood for Zebu animals was 8.7 × 10^3^/μl (±0.46) and for Zebu-Holstein Friesian crossed animals was 9.5 × 10^3^/μl (±0.32), as shown in Table 1.

**Table 1:** White blood cell count (WBC) and differential count for zebu and cross-breeds

S.No		WBC count	Neutrophils count	Monocytes count
	Breed	×10^3^/μl ± SEM	×10^3^/μl (% ± SEM	×10^2^/μl (% ± SEM
1	Zebu (*n* = 6)	8.7 ± 0.46	4.52 (51.9 ± 1.28)	7.8 (8.91 ± 1.35)
2	Cross (*n* = 6)	9.5 ± 0.32	5.61 (58.85 ± 2.55)	4.1 (4.22 ± 1.22)

The table depicts the mean count with standard error of the mean (SEM) for total WBC/μl, total neutrophil count/μl and total monocyte count/μl whole blood. The mean (± SEM) percentage of neutrophils and monocytes among total white cell count are also depicted

### Bovine PMN can be infected by *L. donovani*

**Fig. 1:**
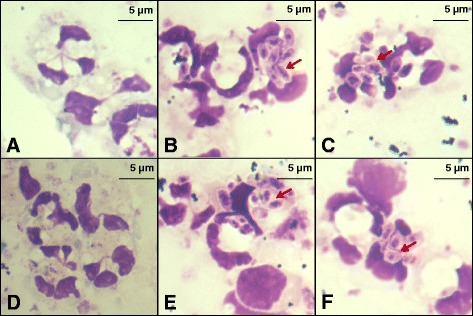
Whole blood and stationary phase promastigotes of *L. donovani* co-incubated for 22 h. Erythrocytes were lysed, and remaining cells were washed twice in PBS (pH 7.2). Cytospin smears from cell suspensions were stained with 10 % Giemsa staining solution, and examined for PMN infection by microscopic observation and enumeration of amastigote like forms inside them. PMN from Zebu-Holstein crossed animals are shown in panels (**a-c**), and from Zebu animals in panels (**d-f**). Panel **a**, **d**: uninfected PMN control; panels **b**, **c**, **d**, **f**: PMN infected with *L. donovani* parasites, designated by the red arrows, at 22 h of culture. The original photomicrographs were taken at 100× magnification

Bovine PMN cells were observed to be infected by *L. donovani* after co-incubation for 22 h; as well amastigote like forms without flagella were observed. Such forms within PMN were observed more frequently at 22 h after infection compared with earlier time points. Not all PMN were equally infected, some PMN harboring many parasites per cell, others had 2–4 parasites per cell and still others with no observable parasites. The various morphology of the PMN is shown in Fig. 1.

**Fig. 2:**
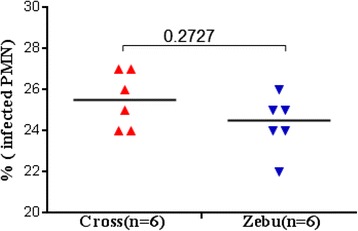
Percentage of *L. donovani-*infected bovine PMN from Zebu or Zebu-Holstein cross-breeds. No statistically significant difference was observed (*P* > 0.05). Bars show the median percentages and *P*-value was calculated using the Mann–Whitney test

Further microscopic examination of cytospin slides revealed a modest difference in *L. donovani* infection rates among PMN between the two cattle breeds, although these differences did not reach statistical significance (Fig. 2). Only 0–1 infected monocytes were observed per 200 PMN counted which would extrapolate to an infection rate of <1 % among all cells present (data not shown).

### Bovine MDM were readily infected with *L. donovani*

**Fig. 3:**
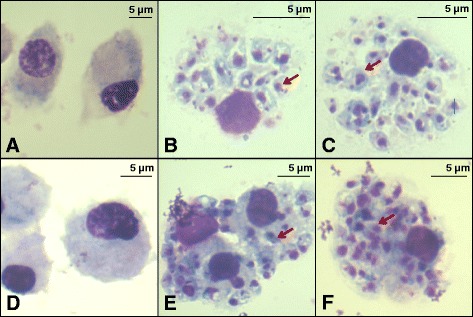
Bovine MDM infection with *L. donovani* stationary promastigotes at day 5 of culture. MDM from either Zebu-Holstein cross-breeds (**a**, **b**, **c**) or Zebu cattle (**d**, **e**, **f**) were either uninfected (panels **a**, **d**) or infected with *L. donovani* (panels **b**, **c**, **e**, **f**). Either uninfected MDM (panels **a**, **d**) or MDM infected with (panels **b**, **c**, **e**, **f**). The original photomicrographs were taken at 100× magnification

At 3 h of incubation, most *L. donovani* stationary stage promastigotes were observed to be attached to MDM via their flagella, whereas only a few were in the posterior polar position. The number of externally attached promastigotes to MDM dramatically decreased after 24 h of co-incubation. At day 5 post-infection the infecting promastigotes had transformed to amastigotes (Fig. 3).

**Fig. 4:**
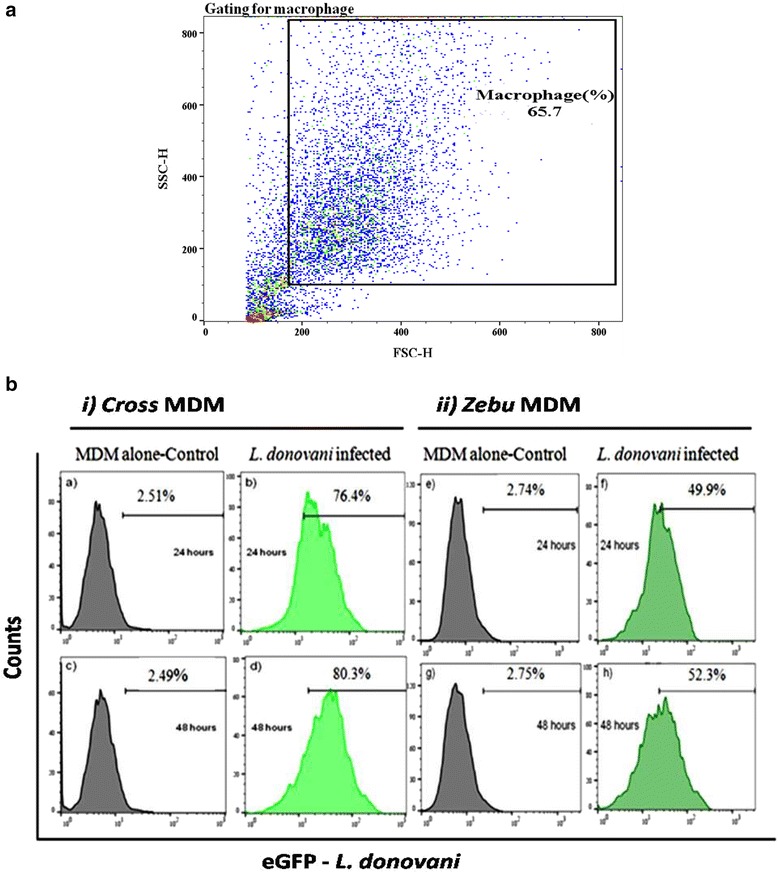
MDM infection with eGFP labelled *L. donovani* was analyzed using the BD FACS Calibur. **a** A representative forward scatter (FSC) and sideward scatter (SSC) plot shows gating for cultured macrophages. **b** A representative infection assay result for eGFP *L. donovani* in bovine MDM at two time points of infection. GFP fluorescence was evaluated among gated macrophages from either cross-breed MDM (i) or Zebu cattle (ii). Grey colored plots represent MDM infected with wild type parasite infections, whereas green colored plots depict MDM infected with eGFP-labelled *L. donovani*. Cells were evaluated after 24 h of infection (*upper panels*) or 48 h of infection (*lower panels*)

Quantitation of eGFP labelled *L. donovani* infection rates by flow cytometry revealed more infected MDM in the cross-breeds, 73.9 % at 24 h, and 77.8 % at 48 h, compared to the MDMs from pure Zebu, 47.16 % at 24 h and 49.55 % at 48 h (Fig. 4).

### Amastigote to promastigote differentiation

Bovine MDM liberated actively motile promastigotes by three  days; these were observed to move in the culture media. At this time most amastigotes in the intact macrophages were seen to actively vibrate. After five days, procycilc promastigotes started to liberate by lysing the MDM and further multiplied in culture. Promastigotes were sub-cultured into liquid media, harvested at log-phase of the culture, and cryopreserved in liquid nitrogen. After one month, cryopreserved *Leishmania* isolates were thawed and sub-cultured in liquid media. All isolates grew well, illustrating their stability during cryopreservation (data not shown).

### Bovine MDM can be infected by *L. donovani* through infected whole blood cells

**Fig. 5:**
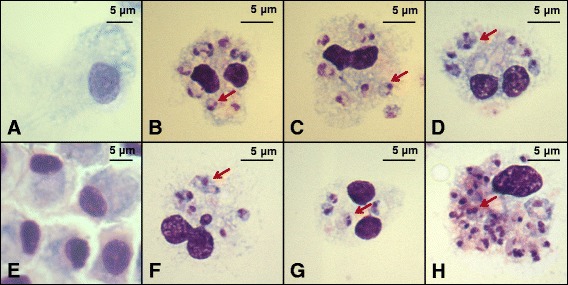
Co-incubation of infected whole blood cells (containing *L. donovani* amastigotes) with MDM successfully induced *Leishmania* infection in the MDM. MDM from cross-breeds (panels **a-d**) or Zebu cattle (panels **e-h**) were mixed with uninfected whole blood (panel **a**, **e**) or whole blood infected with *L. donovani* (panels **b**, **c**, **d**, **f**, **g**, **h**) whole blood. The red arrows depict amastigotes within infected MDM

When bovine MDMs were co-incubated with the infected whole blood cells, MDM infection was apparent confirming that MDM could also be infected through infected bovine whole blood cells (Fig. 5).

### PCR-RFLP confirmed that *L. donovani* parasites infect bovine cells

**Fig. 6:**
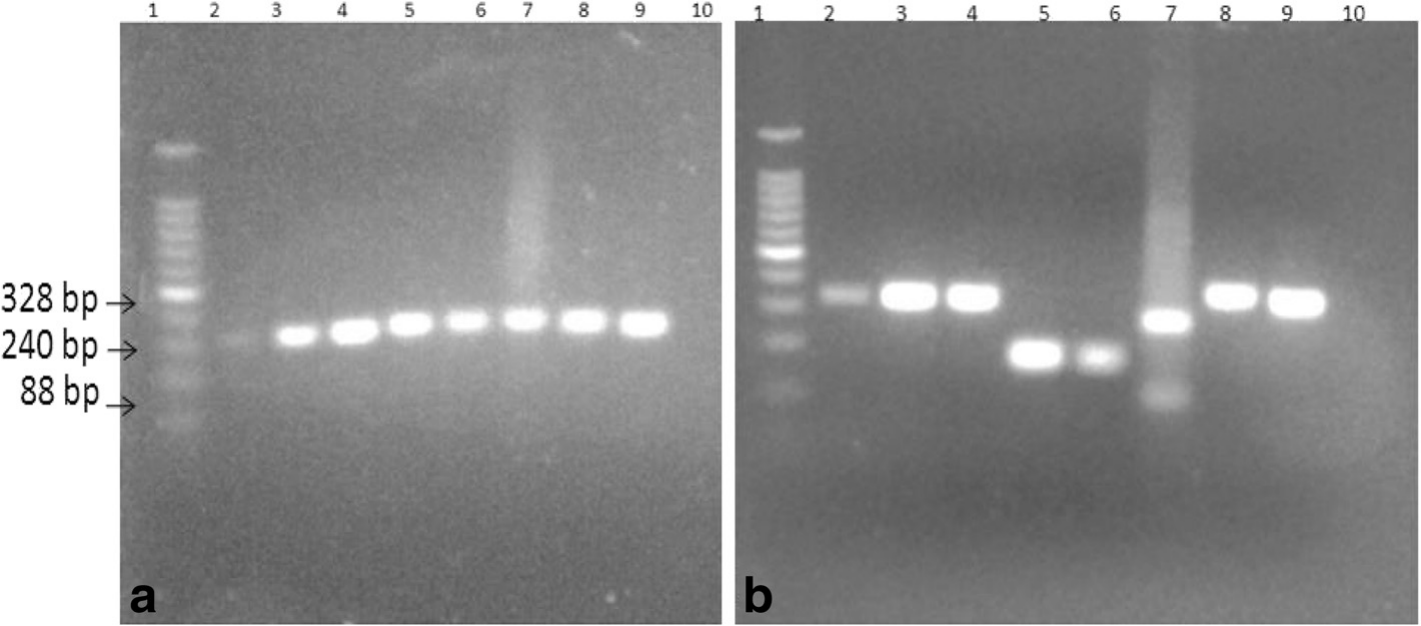
PCR products after ITS-1 primer amplification and *Hha*I digestion of promastigote and amastigote DNA. Panel **a** depicts primer amplification products as follows. Lane 1:100 bp Ladder; Lane 2: *L. donovani* reference strain; Lane 3*: L. donovani* promastigotes retrieved from MDM; Lane 4*: L. donovani* amastigote within MDM; Lane 5: *L. aethiopica* reference strain; Lane 6: *L. aethiopica* culture promastigotes; Lane 7*: L. major*; Lane 8: *L. tropica*; Lane 9: *L. infantum*; Lane10: NC (TE buffer). Panel **b** illustrates *Hha*I-digested ITS-1 amplicons as follows. Lane 1: 100 bp ladder; Lane 2: *L. donovani* reference strain; Lane 3: *L. donovani* cultured promastigotes; Lane 4: *L. donovani* cultured amastigotes; Lane 5: *L. aethiopica* reference strain; Lane 6: *L. aethiopica* culture promastigotes; Lane 7: *L. major*; Lane 8: *L. tropica*; Lane 9: *L. infantum*; Lane 10: NC (TE buffer)

Amplification of parasite DNA with ITS-1 primers resulted in a ~ 328 bp band (Fig. 6a), and the digestion of the PCR products with *Hha*I (Fig. 6b) revealed a 328 bp band for both amastigote and promostigote samples from MDM cultures; a *L. donovani* reference sample resulted in an identical band.

## Discussion

Transmission of VL in Ethiopia is anthroponotic [2]. Recent studies have reported the probable involvement of domestic animals in the transmission of *L. donovani*, which commonly live with humans [8, 9]. None of these studies confirmed live *L. donovani* bodies in cells of these animals or showed in vitro if *L. donovani* parasites could infect the neutrophils and macrophages of these animals. In order to address these issues, a bovine whole blood assay simulating in vivo and in vitro infection of neutrophils and MDM was utilized. To the best of our knowledge, we show for the first time that bovine neutrophils and MDM can be infected when co-incubated with live *L. donovani* promastigotes. These findings support the hypothesis that cattle may serve as reservoir host for *L. donovani* parasites.

Neutrophils are the first cells to infiltrate the site of *Leishmania* promastigote infection, and help to reduce the initial parasite burden [16]. Neutrophils isolated from C57BL/6 mice peritoneal cavities efficiently internalized both amastigote and promastigote forms of the parasite *L. amazonensis* [16].

In mammals, neutrophils are important constituents of innate immune defence involved in phagocytosis [17] and various killing mechanisms to control pathogens [18, 19]. A recent study showed that upon stimulation, bovine neutrophils form neutrophil extracellular traps (NETs), kill bacteria in bovine blood [20]. In vitro studies using human neutrophils showed that *Leishmania* promastigotes can escape neutrophils killing activity [21]. Given the important role of neutrophil-leishmania interactions, there is a need for an appropriate bovine neutrophil assay for leishmania infection.

In this work, we also showed that *L. donovani* stationary promastigotes can infect bovine MDM from both Zebu and Zebu-Holstein Friesian cross-breed cattle, and can be transformed into amastigotes in vitro. The infected bovine MDM were kept in culture for up to 16 days, and were found to maintain amastigotes. It is an established fact that in their preferred mammalian host macrophages *Leishmania* spp. survive and multiply [22]. Experimentally we demonstrated two scenarios of MDM infection: 1) direct infection by stationary phase promastigotes, and 2) infection of MDM following co-culturing of infected whole blood cells. A similar scenario has been documented in *L. major* and other intracellular microbe infections where apoptotic neutrophils are used as a Trojan horse to infect macrophages [23].

Despite the various reports indicating the *L. donovani* seropositivity [24] and isolation of parasite DNA from domestic animals living in VL foci area, and increased human risk to leishmaniasis when living in close proximity [8, 9], there is no evidence indicating the isolation of live parasite from cells of these animals. However, *Leishmania siamensis*, a recently characterised species from outside the subgenera *Leishmania* and *Viannia*, has been isolated from cases of cutaneous bovine leishmaniasis from Switzerland [25]. Similarly, non-typed *Leishmania-*like organism was isolated from skin-infected calf in Zimbabwe [26] and cutaneous leishmaniasis in horses was reported in North America and Puerto Rico [27] where epidemiology of VL due to *L. donovani* is missing.

Naturally, *Leishmania* spp. are obligate intracellular parasites having dimorphic forms, the motile promastigotes form in the sand fly vector, and the non motile intracellular amastigotes form replicating within the macrophages of their mammalian hosts [2]. In our experiment, parasites in the amastigote stages in infected bovine MDM, when placed in fresh NNN/RPMI complete medium at 26 °C for 3–5 days, transformed into motile promastigotes. We could experimentally demonstrate the transformation of amastigotes to promastigotes and *vice versa* using bovine cells in the absence of vectors (sand flies).

Bovine mononuclear phagocyte function plays an important role in natural resistance to another disease model, bovine brucellosis [28]. Moreover, macrophages are known to synthesize natural resistance-associated macrophage protein (Nramp), an integral membrane phosphoglyco protein of 90–100 kDa which is expressed in the lysosomal compartment of macrophages and is rapidly recruited to the membrane of microbe-containing phagosomes formed in these cells [29]. At that site, Nramp functions as a pH-dependent efflux pump for Fe_2_^+^ and Mn_2_^-^, restricting the availability of these essential metals for the engulfed microorganisms and contributing to the antimicrobial defenses of macrophages [30]. In mice, the naturally occurring glycine to aspartic acid mutation at position 169 (G169D) or experimentally induced mutations in Nramp cause susceptibility to a number of infections including *Leishmania* [31], suggesting Nramp plays an essential role in host defence against intracellular pathogens. Genetic polymorphisms in the gene encoding this protein have been reported to contribute to the resistance of African Zebu cattle to bovine tuberculosis [32]. Moreover, a study by Ameni *et al*.[33] with Zebu, Zebu-Holstein crosses, and Holstein cattle under identical field husbandry conditions in Ethiopia, showed both a higher prevalence and severity of bovine TB in the Holstein cattle compared with the Zebu or Zebu-Holstein crosses. However, in our current study of *L. donavani*, only modest differences were observed in susceptibility to infection of MDM among these genetically disparate cattle, although it is possible that these small differences could have reached statistical significance with a larger sample size. Further studies are thus required to ascertain the contribution of genetics in susceptibility of bovine *L. donovani* infections, as has been observed with *M. bovis* [34]. Although we demonstrated that *L. donovani* can infect PMN, we were unable to isolate pure bovine neutrophils to demonstrate if infected neutrophils could be used as a Trojan horse to infect MDM. Morphologically we evaluated the differentiation of bovine monocyte to macrophage under the microscope follow up, however the purity of the MDM population was not determined using surface staining.

## Conclusion

We demonstrated that in vitro *L. donovani* stationary promastigotes infect bovine PMNs and MDM, transform into intracellular amastigote forms which in turn develop into promastigotes upon temperature change to 26 °C. This finding further strengthens the notion that domestic animals could potentially serve as source of infection to the sand fly vectors of visceral leishmaniasis due to *L. donovani*. Given the numbers of Ethiopians residing in VL endemic foci in close association with their cattle, this could represent a significant mechanism for the spread of the disease in the country. However, additional studies are needed to address this possibility.
